# Association between novel per- and poly-fluoroalkyl substances and premature ovarian insufficiency: a case–control study

**DOI:** 10.1093/hropen/hoaf044

**Published:** 2025-07-12

**Authors:** Rui Qiao, Fanghao Guo, Haixia Ding, Di Sun, Qianhui Hu, Yanquan Li, Meiling Zhang, Qing Zhang, Wen Li

**Affiliations:** Center for Reproductive Medicine & Fertility Preservation Program, International Peace Maternity and Child Health Hospital, School of Medicine, Shanghai Jiao Tong University, Shanghai, China; Shanghai Key Laboratory of Embryo Original Diseases, Shanghai 200030, China; Center for Reproductive Medicine & Fertility Preservation Program, International Peace Maternity and Child Health Hospital, School of Medicine, Shanghai Jiao Tong University, Shanghai, China; Shanghai Key Laboratory of Embryo Original Diseases, Shanghai 200030, China; Center for Reproductive Medicine & Fertility Preservation Program, International Peace Maternity and Child Health Hospital, School of Medicine, Shanghai Jiao Tong University, Shanghai, China; Shanghai Key Laboratory of Embryo Original Diseases, Shanghai 200030, China; Center for Reproductive Medicine & Fertility Preservation Program, International Peace Maternity and Child Health Hospital, School of Medicine, Shanghai Jiao Tong University, Shanghai, China; Shanghai Key Laboratory of Embryo Original Diseases, Shanghai 200030, China; Center for Reproductive Medicine & Fertility Preservation Program, International Peace Maternity and Child Health Hospital, School of Medicine, Shanghai Jiao Tong University, Shanghai, China; Shanghai Key Laboratory of Embryo Original Diseases, Shanghai 200030, China; Center for Reproductive Medicine & Fertility Preservation Program, International Peace Maternity and Child Health Hospital, School of Medicine, Shanghai Jiao Tong University, Shanghai, China; Shanghai Key Laboratory of Embryo Original Diseases, Shanghai 200030, China; Center for Reproductive Medicine & Fertility Preservation Program, International Peace Maternity and Child Health Hospital, School of Medicine, Shanghai Jiao Tong University, Shanghai, China; Shanghai Key Laboratory of Embryo Original Diseases, Shanghai 200030, China; Center for Reproductive Medicine & Fertility Preservation Program, International Peace Maternity and Child Health Hospital, School of Medicine, Shanghai Jiao Tong University, Shanghai, China; Shanghai Key Laboratory of Embryo Original Diseases, Shanghai 200030, China; Center for Reproductive Medicine & Fertility Preservation Program, International Peace Maternity and Child Health Hospital, School of Medicine, Shanghai Jiao Tong University, Shanghai, China; Shanghai Key Laboratory of Embryo Original Diseases, Shanghai 200030, China

**Keywords:** premature ovarian insufficiency, novel per- and poly-fluoroalkyl substances (Novel PFAS), sex hormones, reproductive health, mixture models

## Abstract

**STUDY QUESTION:**

Do novel per- and poly-fluoroalkyl substances (Novel PFAS) have associations with premature ovarian insufficiency (POI)?

**SUMMARY ANSWER:**

Hexafluoropropylene oxide dimer acid (HFPO-DA), perfluorobutanoic acid (PFBA), perfluoropentanoic acid (PFPeA), and perfluoropentanesulfonic acid (PFPeS) are associated with an increased risk of POI, and the effect is worse with exposure to mixtures.

**WHAT IS KNOWN ALREADY:**

As public health concerns following Novel PFAS exposure are rising globally, there is a need to understand the exact association between Novel PFAS and various diseases. Epidemiologic studies suggest traditional PFAS exposures adversely affect women’s reproductive health, but the association between exposure to Novel PFAS and POI remains unclear.

**STUDY DESIGN, SIZE, DURATION:**

A retrospective research study, including 371 women, with (case group, n = 151) and without POI (control group, n = 220), was conducted between June 2023 and May 2024.

**PARTICIPANTS/MATERIALS, SETTING, METHODS:**

Thirteen types of Novel PFAS and basal concentrations of FSH, LH, estradiol (E2), and anti-Müllerian hormone (AMH) in plasma samples were measured in plasma samples collected during the early follicular phase (Days 2–5) of a natural menstrual cycle. In addition, characteristics of participants were collected. Both adjusted logistic regression and Bayesian kernel machine regression (BKMR) were used to evaluate associations between Novel PFAS (alone or as a mixture) and POI. Effect modification by age was also investigated.

**MAIN RESULTS AND THE ROLE OF CHANCE:**

The concentrations of HFPO-DA, PFBA, PFPeA, and PFPeS in the case group were significantly higher than in the reference group. The adjusted logistic regression models demonstrated positive associations between plasma concentrations of HFPO-DA, PFBA, PFPeA, and PFPeS with the risk of POI [OR_adj_ = 2.89 (95% CI: 1.84–4.53), 1.54 (95% CI: 1.17–2.02), 3.12 (95% CI: 2.20–4.43), and 2.07 (95% CI: 1.31–3.27), respectively, per 2.7-fold increase in Novel PFAS concentrations]. High concentrations of Novel PFAS showed a negative correlation with AMH and antral follicle count (AFC), but a positive correlation with FSH. After controlling for other covariates, HFPO-DA, PFBA, PFBS, PFPeA, and PFPeS were the major contributors based on the BKMR models.

**LIMITATIONS, REASONS FOR CAUTION:**

False positives cannot be ruled out. Therefore, experiments on PFBA, PFPeA, PFPeS, and HFPO-DA *in vivo* also need to be conducted in animal models.

**WIDER IMPLICATIONS OF THE FINDINGS:**

Our study is the first to discover the impact of Novel PFAS on the incidence of POI, with an investigation of indicators such as AMH, FSH, and AFC. Considering increasingly severe environmental pollution, our research results provide a broader understanding of the impact of environmental endocrine disruptors on ovarian function, and suggest that women of reproductive age should reduce their exposure to Novel PFAS.

**STUDY FUNDING/COMPETING INTEREST(S):**

This work was supported by the National Key Research and Development Project of China (2022YFC2703002), National Natural Science Foundation of China (U24A20658, 82371726), Innovative Research Team of High-Level Local Universities in Shanghai (SHSMU-ZDCX20212200), Shanghai Hospital Development Center Foundation (SHDC22022303, SHDC22022201), and Key project of Medical and Industrial intersection of Shanghai Jiao Tong University (YG2023ZD27), as well as Reproductive Medicine Research Project of the Chinese Red Cross Foundation (HSZH2024GFYZQ) and Open Fund Project of Shanghai Key Laboratory of Embryogenic Diseases (shelab2023ZD02). The funders had no role in the study design, data collection and analysis, decision to publish, or preparation of the manuscript. The authors declare that they have no competing interests.

**TRIAL REGISTRATION NUMBER:**

N/A.

WHAT DOES THIS MEAN FOR PATIENTS? This study looks at whether exposure to certain new types of chemical pollutants, known as ‘Novel PFAS’, might increase the chance of early loss of ovarian function (called premature ovarian insufficiency, or POI). These chemicals are found in everyday places like food, air, soil, and water, meaning many people may come into contact with them. We used advanced research methods to explore how these chemicals, both alone and in combination, might affect ovarian health. Our findings suggest that four specific chemicals (PFBA, HFPO-DA, PFPeA, and PFPeS) are linked to a higher risk of developing POI. We also found that these chemicals may affect important hormones and signs of ovarian function, including anti-Müllerian hormone (AMH), follicle-stimulating hormone (FSH), and the number of small oocyte-containing follicles (AFC). When these pollutants appear together as a mixture, the risk may be even greater.

## Introduction

Currently, the decline in female fertility is a global issue faced worldwide (Sang *et al.*, 2023). Among the numerous factors contributing to this decline, premature ovarian insufficiency (POI) stands out as one of the most severe challenges. The incidence of POI has substantially increased worldwide over recent years. For instance, in North America, the incidence of POI increased from 1% in 2003 to 11.3% in 2023 (95% CI, 9.5–13.1%) among women of reproductive age. A similar trend has also been reported in South America, Asia, and Europe (Li *et al.*, 2023). Previous studies have identified genetic factors as common causes of POI, accounting for 23.5% of cases (Jiao *et al.*, 2018). Iatrogenic and immune factors have also been associated with POI (Ishizuka, 2021; Szeliga *et al.*, 2021; Zhang *et al.*, 2023a). However, these factors are insufficient to explain the rapid increase in POI incidence over the past two decades.

Besides known unmodifiable risk factors for POI (e.g. genetic disorders, autoimmunity), exposure to endocrine-disrupting chemicals (EDC) is a potential contributor to increased POI trends warranting further investigation (Ding *et al.*, 2020; Han *et al.*, 2020). Novel per- and poly-fluoroalkyl substances (Novel PFAS), a new group of persistent EDCs, have been identified in blood and other tissues (Koelmel *et al.*, 2023). Contrary to traditional PFAS [e.g. perfluorooctanoic acid (PFOA) and perfluorooctane sulfonate (PFOS)], Novel PFAS typically include short-chain PFAS (C < 6) and PFAS alternatives [e.g. hexafluoropropylene oxide dimer acid (HFPO-DA), perfluorobutanoic acid (PFBA), and perfluoropentanoic acid (PFPeA)]. Recent studies have reported the detection of Novel PFAS in environmental and biological samples, with both the detection frequency and concentrations exhibiting an upward trend (Kaboré *et al.*, 2022; Zhao *et al.*, 2023). Despite government efforts to control the use of Novel PFAS, plasma concentrations of them in residents decreased by only 28% over a 6-month period, indicating that Novel PFAS exhibit significant bioaccumulation in the human body (Kotlarz *et al.*, 2020). These findings highlight the substantial risk of exposure to Novel PFAS that humanity currently faces.

To date, several studies have reported adverse effects related to these substances. Exposure to PFBA has been shown to inhibit circulatory and nervous system development in zebrafish embryos. HFPO-DA exposure in pregnant Sprague–Dawley rats has been linked to impaired liver function and restricted growth in their offspring. In addition, 6:2 ClPFESA significantly induces cell proliferation and carcinogenicity in HCC cell lines (Conley *et al.*, 2021; Wasel *et al.*, 2022; Hong *et al.*, 2024). The effects of Novel PFAS reported in these studies differ from those of traditional PFAS, underscoring the need for further research into the safety of Novel PFAS. Moreover, population studies have reported that Novel PFAS are associated with a dysfunctional circulatory system (Li *et al.*, 2024), but no regulations or restrictions have been established regarding the exposure concentrations of Novel PFAS in various countries and regions (Lin *et al.*, 2023). Given the rising prevalence of Novel PFAS in the environment, their potential impact on ovarian function is underexplored. The lack of mechanistic studies, particularly their association with the development of primary ovarian insufficiency (POI), warrants more attention than traditional PFAS. To this end, we designed a case–control study to explore the association between Novel PFAS and POI.

In this case–control study, 371 patients (151 with POI vs 220 without POI) under the age of 40 years planning to undergo their first IVF and ICSI were enrolled. Plasma Novel PFAS concentrations are considered the most accurate biomarkers for human Novel PFAS exposure primarily through dietary intake (Guo *et al.*, 2022; Husøy *et al.*, 2023). Our study aims to delineate the detection frequency and concentrations of Novel PFAS in human plasma and to explore their association with POI.

## Materials and methods

### Study design and participants

Between June 2023 and May 2024, at the Reproductive Medicine Center of the IPMCH, 371 infertile patients under the age of 40 years planning to undergo their first IVF and ICSI were enrolled. Women diagnosed with POI were allocated to the case group. The controls were women under the age of 40 years who underwent ART due to tubal factors or male infertility in their partners. Women with conditions that could predispose to reduced ovarian reserve, such as PCOS, autoimmune diseases, a family history of early menopause, prior surgeries, history of radiation or chemotherapy, or other chromosomal or genetic mutations, were excluded from the control group. The diagnosis of POI strictly followed the criteria established by ESHRE: (1) age < 40 years; (2) oligomenorrhea or amenorrhea for at least 4 months; (3) at least two plasma FSH measurements > 25 U/l at an interval of more than 4 weeks; and (4) Subclinical POI was defined as: (i) age < 30 years, anti-Müllerian hormone (AMH) < 1.2 ng/ml, antral follicle count (AFC) < 3–5; (ii) 30 years< age< 40 years, AMH< 1.0 ng/ml, AFC<3 (Szeliga *et al.*, 2021). Patients with other conditions potentially affecting ovarian reserve (polycystic ovary syndrome, history of ovarian surgery, history of chemo- or radio-therapy, endometriosis, and genetic factors, including FOXL2, FMR1, GDF9, BMP15, and Turner syndrome) were excluded from the study. In addition, the age of menopause in the mother of each POI patient included in the study was verified to exclude unknown or undetected genetic-related factors.

### Ethics

This study was approved by the Human Research Ethics Committee of the International Peace Maternity and Child Health Hospital (IPMCH) and received approval on 22 January 2021 (Approval No. [GKLW]2021-18). Participant recruitment was conducted at the Reproductive Medicine Center of IPMCH. Patients were informed about the study’s background, objectives, and procedures by doctors and nurses. Before the study, team members reconfirmed the patients’ willingness to participate and personally provided detailed information about the study.

Moreover, participants had the right to withdraw from the study at any time under any condition without affecting their ongoing or planned IVF or ICSI treatment. The research team committed to protecting the personal information of participants. Patients agreeing to participate in the study were required to sign an informed consent form, acknowledging that they: (1) were familiar with the information about the study; (2) agreed to participate; (3) allowed medical personnel to collect necessary data from their medical records; and (4) agreed to provide blood samples for the study. The research team ensured all participants signed an informed consent form before collecting blood samples.

### Plasma collection and ovarian reserve markers

Plasma samples were collected and measured for ovarian reserve markers, including FSH, LH, estradiol (E2), and AMH (Hao *et al.*, 2023), ∼1 month prior to oocyte retrieval. During routine examinations, the AFC of each patient was assessed during the early follicular phase (Days 2–5) of a natural menstrual cycle. The AFC was defined as the total number of antral follicles (ranging in diameter from 2 to 9 mm) observed in each ovary during the early follicular phase. Furthermore, hormone levels for each patient were determined via a chemiluminescence method.

### Measurement of plasma Novel PFAS

Plasma samples were collected at enrollment, processed immediately, and stored at −80°C until testing. Thirteen Novel PFAS congeners were measured in 100 μl plasma, including perfluoroalkyl carboxylic acids (PFCAs; n = 2), perfluoroalkyl sulfonates (PFSAs; n = 2), and PFAS alternatives (n = 9). Novel PFAS were measured using ultra-performance liquid chromatography coupled with tandem mass spectrometry (UPLC–MS/MS), employing an Agilent 1290 Infinity HPLC system (Agilent Technologies, Inc., Santa Clara, CA, USA) connected to an Agilent 6495C triple quadrupole mass spectrometer (Agilent Technologies), operated in electrospray ionization negative mode (ESI−). Novel PFAS standards were purchased from Wellington Laboratories (Wellington Laboratories Inc., Guelph, ON, Canada). The procedure for determining Novel PFAS has been described in our previous study (Ao *et al.*, 2023). Novel PFAS with a detection frequency below 50% were excluded from the analysis (Table 1).

**Table 1.** Basic information and measured frequencies of Novel PFAS in the case and control groups.

Compound	Full name	LOD	LOQ	Overall (N = 371) [n (%)]	POI cases (N = 151) [n (%)]	Controls (N = 220) [n (%)]
**PFBA**	Perfluorobutanoic acid	0.0025	0.0080	371 (100.0)	151 (100.0)	220 (100.0)
**PFPeA**	Perfluoropentanoic acid	0.0017	0.0054	371 (100.0)	151 (100.0)	220 (100.0)
**PFBS**	Perfluorobutanesulfonic acid	0.0050	0.0159	371 (100.0)	151 (100.0)	220 (100.0)
**PFPeS**	Perfluoropentanesulfonic acid	0.0010	0.0032	371 (100.0)	151 (100.0)	220 (100.0)
**4:2FTS**	4:2 fluorotelomer sulfonic acid	0.0008	0.0026	18 (4.8)	10 (6.6)	8 (3.6)
**HFPO-DA**	Hexafluoropropylene oxide dimer acid	0.0010	0.0032	371 (100.0)	151 (100.0)	220 (100.0)
**ADONA**	4,8-dioxa-3H-perfluorononanoate	0.0003	0.0010	32 (8.6)	10 (6.6)	22 (8.8)
**6:2 FTS**	6:2 fluorotelomer sulfonic acid	0.0030	0.0096	22 (6.0)	9 (5.9)	13 (5.8)
**6:2 ClPFESA**	6:2 chlorinated poly-fluoroethersulfonic acid	0.0008	0.0025	371 (100.0)	151 (100.0)	220 (100.0)
**8:2 FTS**	8:2 fluorotelomer sulfonic acid	0.0038	0.0121	21 (6.6)	13 (8.6)	8 (3.6)
**N-MeFOSAA**	N-methyl perfluorooctane sulfonamidoacetic acid	0.0020	0.0064	23 (6.2)	9 (5.9)	5 (6.3)
**N-EtFOSAA**	N-ethyl perfluorooctane sulfonamidoacetic acid	0.0041	0.0131	9 (2.4)	5 (3.3)	4 (1.8)
**8:2 Cl-PFESA**	8:2 chlorinated poly-fluoroethersulfonic acid	0.0004	0.0012	0 (0.0)	0 (0.0)	0 (0.0)

PFAS, per- and poly-fluoroalkyl substances; LOD, limit of detection, ng/ml; POI, premature ovarian insufficiency; HFPO-DA, hexafluoropropylene oxide dimer acid; PFBA, perfluorobutanoic acid; PFPeA, perfluoropentanoic acid; PFPeS, perfluoropentanesulfonic acid; LOQ, limit of quantification, ng/ml.

### Covariates

The Directed Acyclic Graph for covariate selection in shown in Supplementary Fig. S1. Information about the basic and clinical characteristics of patients was collected from their medical records, and included age, education level, weight (in kilograms), height (in meters), living address, infertility type (primary infertility and secondary infertility), hormone levels [including AMH (in nanograms per milliliter), FSH (in milli-international units per milliliter), LH (in milli-international units per milliliter), and E2 (in picomoles per liter)], and AFC. BMI was calculated as the ratio of an individual’s weight in kilograms divided by the height in meters squared. Education levels included four levels: high school or below, college, university, and master’s degree or above. The living address included three levels: Shanghai urban area, Shanghai suburbs, and ‘other areas’. Infertility types included primary and secondary infertility (Zegers-Hochschild *et al.*, 2017).

### Statistical analyses

We compared the differences between the case and the control group in terms of age, BMI, living address, education level, infertility type, AFC, AMH, FSH, LH, and E2. Continuous variables with a normal distribution were expressed as mean ± SD and then analyzed using Student’s *t*-test. Variables that did not conform to a normal distribution were expressed as Median (interquartile range, IQR) and then analyzed using Mann–Whitney *U* tests. Categorical variables were compared using the chi-square test. The exposure concentration of each chemical in the case and control groups was described with mean±SD and compared using Student’s *t*-test. Concentrations below the limit of quantification (LOQ) were assigned a value equal to one-half the LOQ. Data missing from the analyses were excluded.

Prior to conducting an in-depth analysis, the exposure concentrations of Novel PFAS were natural logarithmic transformed. Multivariate logistic regression models were utilized to estimate the odds ratios (ORs) and 95% CIs for POI in relation to Novel PFAS. Additionally, adjusted ORs and 95% CIs were estimated after accounting for potential confounders, including age (categorized as 25–30, 31–34, and 35–40 years), BMI (<18.5, 18.5–24, and >24 years), education (high school or below, college, university, and master’s degree or above), living address (urban Shanghai, suburban Shanghai, other areas), and infertility type (primary infertility and secondary infertility). Furthermore, we classified plasma Novel PFAS concentrations of the controls into tertiles, with the first tertile serving as a reference, to explore potential dose–response relationships (Supplementary Table S1). To assess the robustness of our results, sensitivity analyses were performed. Given that age is an independent risk factor for POI and that the risk of POI remarkably increased for women ≥35 years of age, we also conducted a subgroup analysis according to age (<35 and ≥35 years).

Pearson coefficients and Multiple linear regression were given to evaluate the correlations among Novel PFAS and between ovarian reserve markers and Novel PFAS. Because of the close correlations among some PFAS, Bayesian kernel machine regression (BKMR) was employed to assess the individual and cumulative effects of Novel PFAS on POI prevalence. BKMR is a Bayesian regression method. It is used to study the effects of multiple environmental exposures on health outcomes. BKMR can model nonlinear relationships and interactions between different pollutants. It uses kernel functions to capture complex patterns among exposures. Unlike traditional linear models, BKMR can show how combined exposures affect health. It works well for analyzing exposure mixtures. The Bayesian framework helps to estimate uncertainty and update results based on the data. BKMR allows researchers to understand both individual and joint effects of exposures. It is useful in environmental health studies with complex data.

Here, to reduce the influence of the variability of different PFAS concentrations, prior to the BKMR analysis, all PFAS concentrations were further normalized after ln-transformation using the *z*-score method. The relative importance of individual Novel PFAS was assessed primarily in two perspectives: the exposure–response relationship and the importance of each Novel PFAS in association with a specific outcome using the posterior inclusion probability (PIP). Estimates of BKMR were generated after 20 000 iterations. R statistical software (Version 4.3.2; R Foundation for Statistical Computing, Vienna, Austria) was applied for all analyses. Statistical significance was determined at a two-tailed *P* < 0.05.

## Results

### Study population characteristics

A total of 371 women were included in the final analysis, including 151 women with POI (case group) and 220 women without POI (control group). Characteristics and biomarkers of ovarian reserve of the two groups are summarized in Table 2. The ages, BMIs, living addresses, education levels, infertility types, and LH and E2 levels were similar between the two groups. Compared with controls, women with POI had lower AFCs and lower levels of AMH, but higher levels of FSH.

**Table 2.** Characteristics of infertile women with and without POI: a case–control study conducted in Shanghai during 2023–2024.

	Overall (N = 371) [mean±SD, n (%), Median (IQR)]	POI cases (N = 151) [mean±SD, n (%), Median (IQR)]	Controls (N = 220) [mean±SD, n (%), Median (IQR)]	*P-*value
**Age**				
Mean±SD	34.06 ± 3.18	34.13 ± 3.32	33.83 ± 3.83	0.353
≤30	51 (14.0)	15 (9.9)	36 (1.7)	0.129
30–34	135 (36.3)	50 (33.1)	85 (38.5)	–
≥35	185 (49.7)	86 (57.0)	99 (44.8)	–
**BMI (kg/m^2^)**				
Mean±SD	22.15 ± 2.97	22.42 ± 2.81	22.38 ± 3.10	0.905
<18.5	30 (8.3)	9 (6.0)	21 (10.0)	0.118
18.5–24	223 (59.9)	82 (54.3)	141 (63.8)	–
>24	118 (31.8)	60 (39.7)	58 (26.2)	–
**Living address**				
Shanghai urban area	77 (21.0)	27 (17.9)	50 (23.1)	0.333
Shanghai suburb area	60 (16.1)	20 (13.2)	40 (18.1)	–
Other area	234 (62.9)	104 (68.9)	130 (58.8)	–
**Education**				
High school or below	69 (18.8)	26 (17.2)	43 (19.9)	0.974
College	70 (18.8)	28 (18.5)	42 (19.0)	–
University	197 (53.0)	83 (55.0)	114 (51.6)	–
Master’s degree or above	35 (9.4)	14 (9.3)	21 (9.5)	–
**Infertility type**				
Primary infertility	200 (54.0)	88 (58.2)	112 (51.1)	0.320
Secondary infertility	171 (46.0)	63 (41.8)	108 (48.9)	–
**AFC**	7.42 ± 2.81	3.21 ± 1.83	11.90 ± 3.89	<0.001
**AMH**	1.35 ± 2.81	0.59 ± 0.35	3.52 ± 1.12	<0.001
**FSH**	7.87 ± 6.81	13.67 ± 5.71	6.79 ± 1.66	<0.001
**LH**	3.90 (2.46–6.00)	3.40 (2.20–5.78)	4.10 (3.10–6.07)	0.215
**E2**	133.1 (100.1–189.1)	126.60 (89.10–180.50)	135.00 (108.00–195.50)	0.710

–, not applicable; Age, year; AFC, antral follicle count/number; AMH, anti-Müllerian hormone, ng/ml; E2, estradiol, pmol/ml; POI, Premature ovarian insufficiency; IQR, interquartile range. Mean±SD were compared using Student’s *t*-test; IQR were compared using the Mann–Whitney *U* test; n (%) were compared using the chi-square test.

### Concentrations of Novel PFAS

PFBS, perfluoropentanesulfonic acid (PFPeS), PFBA, PFPeA, 6:2 ClPFESA, and HFPO-DA were detected in 100% of the samples (Table 1). The concentrations of HFPO-DA (0.021 ± 0.026 vs 0.011 ± 0.005, *P* < 0.001), PFBA (0.084 ± 0.121 vs 0.038 ± 0.035, *P* < 0.001), PFPeA (0.044 ± 0.122 vs 0.007 ± 0.006, *P* < 0.001), and PFPeS (0.021 ± 0.028 vs 0.012 ± 0.009, *P *< 0.001) in the case group were significantly higher than those in the control group. No significant difference was found for the concentrations of 6:2 ClPFESA and PFBS (Table 3).

**Table 3.** Plasma concentrations of Novel PFAS for women with and without POI.

Compound	Overall (N = 371) [Mean±SD]	POI cases (N = 151) [Mean±SD]	Controls (N = 220) [Mean±SD]	*P-*value
**PFBA**	0.057 ± 0.085	0.084 ± 0.121	0.038 ± 0.035	<0.001
**PFPeA**	0.022 ± 0.080	0.044 ± 0.122	0.007 ± 0.006	<0.001
**PFBS**	0.152 ± 0.128	0.144 ± 0.157	0.150 ± 0.148	0.416
**PFPeS**	0.016 ± 0.019	0.021 ± 0.028	0.012 ± 0.009	<0.001
**HFPO-DA**	0.014 ± 0.018	0.021 ± 0.026	0.011 ± 0.005	<0.001
**6:2 ClPFESA**	2.401 ± 2.531	2.272 ± 2.091	2.482 ± 2.801	0.824

Note: The unit for each chemical is ng/ml; Mean±SD were compared using Student’s *t*-test.

PFAS, per- and poly-fluoroalkyl substances; POI: premature ovarian insufficiency; 6:2 ClPFESA, 6:2 chlorinated poly-fluoroethersulfonic acid; HFPO-DA, hexafluoropropylene oxide dimer acid; PFBA, perfluorobutanoic acid; PFBS,  perfluorobutanesulfonic acid; PFPeA, perfluoropentanoic acid; PFPeS, perfluoropentanesulfonic acid.

### Novel PFAS and POI

In the adjusted logistic regression models, we found positive associations between plasma concentrations of HFPO-DA, PFBA, PFPeA, and PFPeS with the risk of POI [OR_adj_=2.89 (95% CI: 1.84–4.53), 1.54 (95% CI: 1.17–2.02), 3.12 (95% CI: 2.20–4.43), and 2.07 (95% CI: 1.31–3.27), respectively, per 2.7-fold increase in plasma concentrations] (Table 4). Additionally, in stratified analyses, similar results were observed among women with ages <35 and ≥35 years (Supplementary Tables S2 and S3). We also stratified the exposure concentrations based on the IQR of the six Novel PFAS and constructed a multivariate logistic regression model (Supplementary Table S4). However, we found that an increase in PFBS concentration was associated with a decrease in the risk of POI. The results of the other subgroup analyses were consistent with those of the overall analysis, indicating that increased exposure concentrations of HFPO-DA and PFPeA significantly elevate the risk of developing POI.

**Table 4.** Multivariate logistic regression models for the associations of Novel PFAS with POI: a case–control study conducted in Shanghai during 2023–2024 (n = 371).

**Compound** ^#^	Crude OR	Crude OR *P*-value	**Adjusted OR** ^§^	**Adjusted OR *P*-value** ^§^
**PFBA**	1.43 (1.11–1.84)	0.005	1.54 (1.17–2.02)	0.002
**PFPeA**	3.20 (2.29–4.47)	<0.001	3.12 (2.20–4.43)	<0.001
**PFBS**	0.56 (0.38–0.83)	0.004	0.57 (0.37–0.86)	0.007
**PFPeS**	1.89 (1.25–4.47)	0.003	2.07 (1.31–3.27)	0.002
**HFPO-DA**	2.72 (1.78–4.16)	<0.001	2.89 (1.84–4.53)	<0.001
**6:2 ClPFESA**	0.95 (0.72–1.25)	0.705	0.98 (0.73–1.31)	0.897

Note:

# The concentrations of Novel PFAS were natural logarithmic transformed.

§ Multivariate logistic regression model with adjustments of age, BMI, education, infertility type, and living address.

PFAS, per- and poly-fluoroalkyl substances; POI: premature ovarian insufficiency; OR: odds ratio; 6:2 ClPFESA, 6:2 chlorinated poly-fluoroethersulfonic acid; HFPO-DA, hexafluoropropylene oxide dimer acid; PFBA, perfluorobutanoic acid; PFPeA, perfluoropentanoic acid; PFPeS, perfluoropentanesulfonic acid.

### Ovarian reserve markers and Novel PFAS

According to adjusted multiple linear regression, the plasma concentration of PFPeA in patients with POI were positively associated with FSH levels [*β*_adj_=0.77 (95% CI: 0.23–1.30), per 2.7-fold increase in plasma concentrations] and negatively associated with E2 levels [*β*_adj_=−1.38 (95% CI: −1.82 to −0.95), per 2.7-fold increase in plasma concentrations] (Table 5). Moreover, PFBA, HFPO-DA, and PFPeS also maintained similar associations with FSH and AFC.

**Table 5.** Associations between Novel PFAS and ovarian reserve markers in patients with POI (n = 371).

**Compound** ^#^		AMH	AFC	FSH	E2	LH
**PFBA**	**Crude *β* (95% CI)**	−0.09 (−0.33 to 0.03)	−0.19 (−0.61 to 0.23)	0.40 (−0.10 to 0.89)	−4.57 (−19.45 to 10.31)	0.09 (−0.16 to 0.35)
	^§^ **Adjusted *β* (95% CI)**	−0.11 (−0.36 to −0.10)*	−0.31 (−0.72 to −0.11)	0.44 (−0.05 to 0.94)	−4.50 (−19.45 to 10.43)	−0.06 (−0.19 to 0.32)
**PFPeA**	**Crude *β* (95% CI)**	−0.24 (−0.64 to −0.27)***	−1.56 (−2.00 to −1.12)***	0.87 (0.34–1.39)***	1.20 (−14.42 to 16.81)	−0.06 (−0.33 to 0.20)
	^§^ **Adjusted *β* (95% CI)**	−0.22 (−0.60 to −0.23)***	−1.38 (−1.82 to −0.95)***	0.77 (0.23–1.30)***	2.93 (−12.85 to 10.70)	−0.05 (−0.32 to 0.21)
**PFBS**	**Crude *β* (95% CI)**	0.11 (0.43–0.61)*	1.06 (0.38–1.73)*	−0.40 (−1.20 to 0.39)	−1.59 (−25.53 to 22.35)	−0.12 (−0.52 to 0.29)
	^§^ **Adjusted *β* (95% CI)**	0.10 (0.01–0.56)*	0.94 (0.28–1.60)*	−0.33 (−1.12 to 0.47)	−4.80 (−27.79 to 19.77)	−0.11 (0.51–0.30)
**PFPeS**	**Crude *β* (95% CI)**	−0.13 (−0.65 to −0.09)*	−1.28 (−1.95 to −0.62)*	0.72 (−0.06 to 1.50)	12.46 (−11.14 to 36.06)	0.03 (−0.37 to 0.43)
	^§^ **Adjusted *β* (95% CI)**	−0.12 (−0.61 to −0.15)***	−1.33 (−2.00 to −0.67)**	0.84 (0.48–1.63)**	13.34 (−10.53 to 37.21)	−0.02 (−0.40 to 0.42)
**HFPO-DA**	**Crude *β* (95% CI)**	−0.15 (−0.68 to −0.14)**	−0.99 (−1.63 to −0.34)**	0.67 (−0.09 to 1.41)	−15.46 (−38.09 to 7.17)	−0.17 (− 0.55 to 0.21)
	^§^ **Adjusted *β* (95% CI)**	−0.13 (−0.61 to −0.07)**	−0.89 (−1.52 to −0.25)**	0.62 (−0.13 to 1.38)	−9.34 (−32.00 to 13.31)	0.10 (−0.48 to 0.29)
**6:2 ClPFESA**	**Crude *β* (95% CI)**	0.01 (−0.19 to 0.21)	0.06 (−0.43 to 0.54)	−0.53 (−1.09 to 0.42)	1.46 (−15.70 to 18.61)	0.21 (−0.08 to 0.50)
	^§^ **Adjusted *β* (95% CI)**	−0.01 (−0.21 to 0.18)	0.04 (−0.44 to 0.51)	−0.55 (−1.12 to 0.22)	3.42 (−13.69 to 20.53)	0.24 (−0.05 to 0.53)

Note:

# The concentrations of Novel PFAS were natural logarithmic transformed.

§ Multiple linear regression model with adjustments of age, BMI, education, infertility type, and living address.

* *P *< 0.05.

** *P *< 0.01.

*** *P *< 0.001.

PFAS, per- and poly-fluoroalkyl substances; POI, premature ovarian insufficiency; 6:2 ClPFESA, 6:2 chlorinated poly-fluoroethersulfonic acid; HFPO-DA, hexafluoropropylene oxide dimer acid; PFBA, perfluorobutanoic acid; PFBS,  perfluorobutanesulfonic acid; PFPeA, perfluoropentanoic acid; PFPeS, perfluoropentanesulfonic acid; AMH, anti-Müllerian hormone; E2, estradiol, pmol/ml; AFC, antral follicle count.

### Results of Novel PFAS joint and individual exposures in BKMR analyses

**Figure 1.**
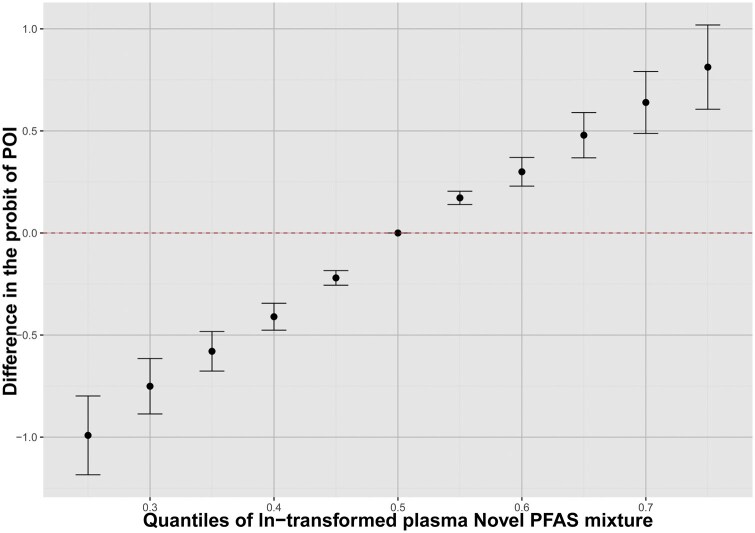
**Overall joint associations of the Novel PFAS mixture in infertile women diagnosed with POI**. Overall joint associations of the Novel PFAS mixture (estimates and 95% CIs) in infertile women diagnosed with POI estimated by Bayesian kernel machine regression (BKMR) (n=371). All estimates were adjusted for age, BMI, education, infertility type, and living address. This figure plots the estimated difference in the probit of POI when exposures are at a particular percentile (*x*-axis) in comparison with when exposures are all at the 50th percentile. POI, premature ovarian insufficiency; PFAS, per- and poly-fluoroalkyl substances.

**Figure 2.**
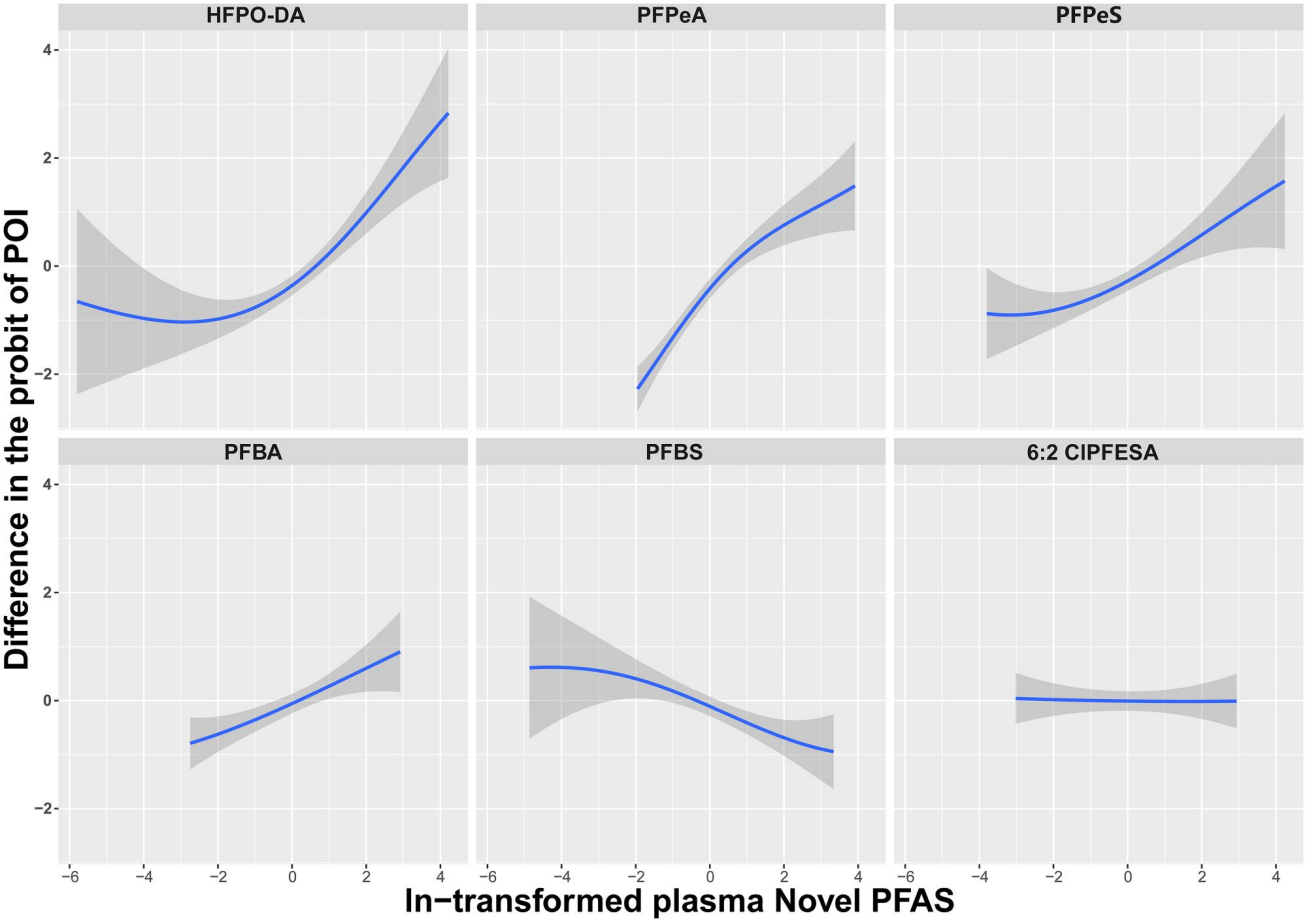
**Univariate exposure–response relationship of individual plasma Novel PFAS concentrations in infertile women diagnosed with POI**. Univariate exposure–response relationship of individual plasma Novel PFAS concentrations (estimates and 95% CIs) in Chinese women diagnosed with POI estimated by Bayesian kernel machine regression (BKMR) for each Novel PFAS, with the other pollutants fixed at the median (n=371). All estimates were adjusted for age, BMI, education, infertility type, and living address. The boundaries of the gray areas represent the 95% CIs of the exposure–response relationship. For full chemical names, see Table 1. POI, premature ovarian insufficiency; PFAS, per- and poly-fluoroalkyl substance.

A clustered heatmap of the correlations between the concentrations of the Novel PFAS is shown in Supplementary Fig. S2. Figure 1 shows the relationship between the quantiles of plasma Novel PFAS mixture in women and the difference in the probit of POI risk. As the PFAS concentrations in the plasma increases, the difference in the probit of POI risk gradually rises, indicating that higher PFAS exposure concentrations may be significantly associated with an increased risk of POI. At lower concentrations of PFAS exposure, the difference in the probit of POI risk is close to 0, suggesting that exposure has a minimal impact on POI risk. However, as the PFAS concentration increases, especially at higher quantiles, the difference in the probit of POI risk significantly increases, indicating that higher PFAS exposures may negatively affect ovarian function and increase the risk of POI. Figure 2 and Supplementary Fig. S3 present the relationship between the quantiles of plasma concentrations of Novel PFAS and the differences in the probit of POI risk. These findings suggest that Novel PFAS, such as HFPO-DA and PFPeA, are more strongly associated with the risk of POI than the others. There were no significant interactions between PFAS homologs (Supplementary Fig. S4).

## Discussion

PFAS represent a persistent global pollution challenge (Evich *et al.*, 2022). With the growing restrictions on traditional PFAS, there is a rise in the use of novel PFAS, highlighting the need for in-depth investigation (Spyrakis and Dragani, 2023). Our study highlights that Novel PFAS are increasingly detected in the population and are closely linked to the incidence of POI. Increased exposure to Novel PFAS significantly elevates the risk of POI and is closely related to AMH, AFC, and FSH.

In this work, a higher detection frequency of Novel PFAS: PFBA, PFPeA, PFPeS, and HFPO-DA was found in plasma, probably induced by the gradual prohibition of traditional PFAS and the increasing use of Novel PFAS (DeLuca *et al.*, 2022). However, the concentrations of Novel PFAS are not as high as traditional PFAS in plasma. The median and IQR of PFOA in women preconception in Shanghai, China (2013–2015, n = 950) were reported to be 13.84 (10.08–18.83) ng/ml (Zhou *et al.*, 2017). The median and IQR of PFOA in women in Nanjing, China (2013–2016, n = 240) were reported to be 11.10 (7.6–14.45) ng/ml (Zhang *et al.*, 2018). The median and IQR of PFOA in women preconception in Shanghai, China were reported to be 9.79 (7.63–12.43) ng/ml in 2023, decreasing at a rate of 30% in recent decades (Mao *et al.*, 2024). This suggests that Novel PFAS may reach exposure concentrations comparable to PFOA in the future. However, there are no policies or regulations in place to limit exposure to these compounds. This situation is undoubtedly worrisome. This study is also the first to report positive associations of PFBA, PFPeA, PFPeS, and HFPO-DA with risk of POI. In our study, the six most frequently detected Novel PFAS exhibited low correlations with each other, with most correlation coefficients ranging between 0.1 and 0.3, indicating weak collinearity. Thus, we constructed a mixed model to explore their joint associations with POI, revealing that the Novel PFAS mixture was positively associated with POI.

However, it is currently unclear how Novel PFAS are involved in the development of POI. Disruptions to endocrine function and hindrances in follicular development could be factors contributing to this process. We found that these PFAS are associated with higher levels of FSH and lower levels of AFC and AMH, indicating that Novel PFAS may disrupt the physiological functions of endocrine cells in the ovary and could potentially act as EDCs (Neff *et al.*, 2022). This is similar to some previous studies focusing on traditional PFAS. In 2018, a study reported that increased exposure concentrations of PFOA and PFOS significantly raise the risk of POI and are significantly negatively correlated with AMH levels (Zhang *et al.*, 2018). Several epidemiological studies have shown a significant positive correlation between exposure concentrations of PFOA (and PFOS) and FSH levels (Luo *et al.*, 2021; Rodríguez-Carrillo *et al.*, 2023), which is consistent with our findings. Another epidemiological study has shown a significant negative correlation between PFBS exposure concentrations and FSH levels in the population (Nian *et al.*, 2020). In *in vivo* studies, prepubertal female rats exposed to PFDoA exhibited suppressed mRNA expression of Lhcgr, Star, and Hsd17b3, ultimately leading to reduced E2 levels (Shi *et al.*, 2009). *In vitro* studies have reported that PFOS activates the PPAR-γ signaling pathway, and inhibits the secretion levels of LH and E2 in ovarian granulosa cells (Chaparro-Ortega *et al.*, 2018). These differences may be caused by the substantial gap between experimental modeling and actual human exposure. Previous studies have primarily employed high-dose, short-term exposure methods, and even the lower-dose exposure methods reported so far still far exceed legally prescribed limits (Zhang *et al.*, 2023b). Human exposure occurs over decades, with gradual accumulation. Moreover, studies on Novel PFAS have predominantly focused on their effects on the liver, with little attention given to their impact on ovarian function (Conley *et al.*, 2021). Our study is the first to report significant harm caused by Novel PFAS to human ovarian endocrine function. Even though current exposure concentrations are relatively low, with continued accumulation, Novel PFAS could reach exposure concentrations comparable to those of PFOA and other PFAS in the coming decades. Therefore, our findings are undoubtedly of great significance.

The toxic effects of Novel PFAS on oocytes and follicular development are not well understood. Oocytes are exposed to follicular fluid, which is primarily composed of fluids secreted by surrounding blood vessels. Therefore, plasma concentrations of Novel PFAS can represent the concentrations of Novel PFAS in follicular fluid (Kang *et al.*, 2020). Few studies have focused specifically on Novel PFAS and oocytes or follicles. However, according to studies on traditional PFAS, the apoptotic pathway may be one of the important mechanisms by which PFAS affect the development of oocytes and follicles (Hallberg *et al.*, 2019; Ding *et al.*, 2020; Leclercq *et al.*, 2022). *In vitro* studies have shown that PFOA and PFNA can induce apoptosis in ovarian granulosa cells by interfering with mitochondrial function and inducing oxidative stress, leading to developmental and maturation disorders in oocytes (Hallberg *et al.*, 2019; Jiao *et al.*, 2021). Animal studies have found that even low-dose chronic exposure to PFOA can induce apoptosis in ovarian granulosa cells, leading to a reduction in ovarian reserve in adult female mice (Zhang *et al.*, 2023b). Additionally, PFOA has been reported to disrupt the genetic material of oocytes, leading to developmental disorders (Estefanía González-Alvarez *et al.*, 2022). Although direct evidence of Novel PFAS affecting follicular development is currently lacking, a few studies suggest that Novel PFAS may have adverse effects on the development of zebrafish and rats (Conley *et al.*, 2021; Wasel *et al.*, 2022). Therefore, we believe that further exploration of the association between Novel PFAS and reproductive health has great scientific and social significance, especially in terms of potential mechanisms.

In all, our study focused on exploring the relationship between the concentrations of Novel PFAS in women’s plasma and POI for the first time. Our research introduces epidemiological evidence of a novel link between Novel PFAS and POI incidence, addressing gaps in current studies, highlighting the reproductive toxicity of novel PFAS, and providing insights into POI mechanisms. However, limitations also remain. First, due to its case–control design, a causal inference between Novel PFAS exposure and POI cannot be established. Second, although we observed high detection frequencies of Novel PFAS, there is currently a lack of systematic data on their temporal trends in human tissues, making it difficult to assess whether bodily burdens are increasing over time. Third, the relatively low serum concentrations of Novel PFAS compared to traditional PFAS raise the possibility of residual confounding or indirect associations, especially given the widespread and correlated nature of environmental chemical exposures. Finally, the biological mechanisms underlying the observed associations remain unclear. Future studies, including prospective cohort designs, longitudinal biomonitoring, and mechanistic *in vitro* and *in vivo* experiments, are needed to validate our findings and elucidate causal pathways.

## Conclusion

In this study, we investigated the relationship between Novel PFAS and POI. We demonstrated that high concentrations of PFPeA, HFPO-DA, PFBA, and PFPeS are associated with a greater risk of POI and its related indicators. Moreover, high concentrations of Novel PFAS showed a negative correlation with AMH and AFC, but a positive correlation with FSH. Furthermore, our findings highlight the potential reproductive risks associated with increasing exposure to Novel PFAS, suggesting that greater attention should be paid to their possible impact on ovarian reserve and female fertility.

## Supplementary Material

hoaf044_Supplementary_Data

## Data Availability

All data are available in the main text or the supplementary materials.

## References

[hoaf044-B1] Ao Y , Nian M, Tang W, Zhang J, Zhang Q, Ao J. A sensitive and robust method for the simultaneous determination of thirty-three legacy and emerging per- and polyfluoroalkyl substances in human plasma and serum. Anal Bioanal Chem 2023;415:457–470. 36383228 10.1007/s00216-022-04426-4

[hoaf044-B2] Chaparro-Ortega A , Betancourt M, Rosas P, Vázquez-Cuevas FG, Chavira R, Bonilla E, Casas E, Ducolomb Y. Endocrine disruptor effect of perfluorooctane sulfonic acid (PFOS) and perfluorooctanoic acid (PFOA) on porcine ovarian cell steroidogenesis. Toxicol In Vitro 2018;46:86–93. 28982594 10.1016/j.tiv.2017.09.030

[hoaf044-B3] Conley JM , Lambright CS, Evans N, McCord J, Strynar MJ, Hill D, Medlock-Kakaley E, Wilson VS, Gray LE. Hexafluoropropylene oxide-dimer acid (HFPO-DA or GenX) alters maternal and fetal glucose and lipid metabolism and produces neonatal mortality, low birthweight, and hepatomegaly in the Sprague-Dawley rat. Environ Int 2021;146:106204. 33126064 10.1016/j.envint.2020.106204 PMC7775906

[hoaf044-B4] DeLuca NM , Minucci JM, Mullikin A, Slover R, Cohen Hubal EA. Human exposure pathways to poly- and perfluoroalkyl substances (PFAS) from indoor media: a systematic review. Environ Int 2022;162:107149. 35240384 10.1016/j.envint.2022.107149 PMC11577573

[hoaf044-B5] Ding N , Harlow SD, Randolph JF, Loch-Caruso R, Park SK. Perfluoroalkyl and polyfluoroalkyl substances (PFAS) and their effects on the ovary. Hum Reprod Update 2020;26:724–752. 32476019 10.1093/humupd/dmaa018 PMC7456353

[hoaf044-B6] Estefanía González-Alvarez M , Severin A, Sayadi M, Keating AF. PFOA-induced ovotoxicity differs between lean and obese mice with impacts on ovarian reproductive and DNA damage sensing and repair proteins. Toxicol Sci 2022;190:173–188. 36214631 10.1093/toxsci/kfac104 PMC9789752

[hoaf044-B7] Evich MG , Davis MJB, McCord JP, Acrey B, Awkerman JA, Knappe DRU, Lindstrom AB, Speth TF, Tebes-Stevens C, Strynar MJ et al Per- and polyfluoroalkyl substances in the environment. Science 2022;375:eabg9065. 35113710 10.1126/science.abg9065 PMC8902460

[hoaf044-B8] Guo P , Furnary T, Vasiliou V, Yan Q, Nyhan K, Jones DP, Johnson CH, Liew Z. Non-targeted metabolomics and associations with per- and polyfluoroalkyl substances (PFAS) exposure in humans: a scoping review. Environ Int 2022;162:107159. 35231839 10.1016/j.envint.2022.107159 PMC8969205

[hoaf044-B9] Hallberg I , Kjellgren J, Persson S, Örn S, Sjunnesson Y. Perfluorononanoic acid (PFNA) alters lipid accumulation in bovine blastocysts after oocyte exposure during in vitro maturation. Reprod Toxicol 2019;84:1–8. 30502403 10.1016/j.reprotox.2018.11.005

[hoaf044-B10] Han C , Wei Y, Geng Y, Cui Y, Li S, Bao Y, Shi W. Bisphenol A in utero exposure induces ovary dysfunction in mice offspring and the ameliorating effects of Cuscuta chinensis flavonoids. Environ Sci Pollut Res Int 2020;27:31357–31368. 32488702 10.1007/s11356-020-09202-4

[hoaf044-B11] Hao Y , Wang Y, Yan L, Xu X, Chen D, Zhao Y, Qiao J. Synthetic phenolic antioxidants and their metabolites in follicular fluid and association with diminished ovarian reserve: a case-control study. Environ Health Perspect 2023;131:67005. 37267061 10.1289/EHP11309 PMC10237312

[hoaf044-B12] Hong J , Du K, Jin H, Chen Y, Jiang Y, Zhang W, Chen D, Zheng S, Cao L. Evidence of promoting effects of 6:2 Cl-PFESA on hepatocellular carcinoma proliferation in humans: an ideal alternative for PFOS in terms of environmental health? Environ Int 2024;186:108582. 38513556 10.1016/j.envint.2024.108582

[hoaf044-B13] Husøy T , Caspersen IH, Thépaut E, Knutsen H, Haug LS, Andreassen M, Gkrillas A, Lindeman B, Thomsen C, Herzke D et al Comparison of aggregated exposure to perfluorooctanoic acid (PFOA) from diet and personal care products with concentrations in blood using a PBPK model—results from the Norwegian biomonitoring study in EuroMix. Environ Res 2023;239:117341. 37839534 10.1016/j.envres.2023.117341

[hoaf044-B14] Ishizuka B. Current understanding of the etiology, symptomatology, and treatment options in premature ovarian insufficiency (POI). Front Endocrinol (Lausanne) 2021;12:626924. 33716979 10.3389/fendo.2021.626924 PMC7949002

[hoaf044-B15] Jiao X , Ke H, Qin Y, Chen Z-J. Molecular genetics of premature ovarian insufficiency. Trends Endocrinol Metab 2018;29:795–807. 30078697 10.1016/j.tem.2018.07.002

[hoaf044-B16] Jiao X , Liu N, Xu Y, Qiao H. Perfluorononanoic acid impedes mouse oocyte maturation by inducing mitochondrial dysfunction and oxidative stress. Reprod Toxicol 2021;104:58–67. 34246765 10.1016/j.reprotox.2021.07.002 PMC8477654

[hoaf044-B17] Kaboré HA , Goeury K, Desrosiers M, Vo Duy S, Liu J, Cabana G, Munoz G, Sauvé S. Novel and legacy per- and polyfluoroalkyl substances (PFAS) in freshwater sporting fish from background and firefighting foam impacted ecosystems in Eastern Canada. Sci Total Environ 2022;816:151563. 34762942 10.1016/j.scitotenv.2021.151563

[hoaf044-B18] Kang Q , Gao F, Zhang X, Wang L, Liu J, Fu M, Zhang S, Wan Y, Shen H, Hu J. Nontargeted identification of per- and polyfluoroalkyl substances in human follicular fluid and their blood-follicle transfer. Environ Int 2020;139:105686. 32278886 10.1016/j.envint.2020.105686

[hoaf044-B19] Koelmel JP , Lin EZ, Parry E, Stelben P, Rennie EE, Godri Pollitt KJ. Novel perfluoroalkyl substances (PFAS) discovered in whole blood using automated non-targeted analysis of dried blood spots. Sci Total Environ 2023;883:163579. 37100129 10.1016/j.scitotenv.2023.163579 PMC10247435

[hoaf044-B20] Kotlarz N , McCord J, Collier D, Lea CS, Strynar M, Lindstrom AB, Wilkie AA, Islam JY, Matney K, Tarte P et al Measurement of novel, drinking water-associated PFAS in blood from adults and children in Wilmington, North Carolina. Environ Health Perspect 2020;128:77005. 32697103 10.1289/EHP6837 PMC7375159

[hoaf044-B21] Leclercq A , Ranefall P, Sjunnesson YCB, Hallberg I. Occurrence of late-apoptotic symptoms in porcine preimplantation embryos upon exposure of oocytes to perfluoroalkyl substances (PFASs) under in vitro meiotic maturation. PLoS One 2022;17:e0279551. 36576940 10.1371/journal.pone.0279551 PMC9797085

[hoaf044-B22] Li M , Zhu Y, Wei J, Chen L, Chen S, Lai D. The global prevalence of premature ovarian insufficiency: a systematic review and meta-analysis. Climacteric 2023;26:95–102. 36519275 10.1080/13697137.2022.2153033

[hoaf044-B23] Li S , Wang C, Yang C, Chen Y, Cheng Q, Liu J, Zhang Y, Jin L, Li Z, Ren A et al Prenatal exposure to poly/perfluoroalkyl substances and risk for congenital heart disease in offspring. J Hazard Mater 2024;469:134008. 38503211 10.1016/j.jhazmat.2024.134008

[hoaf044-B24] Lin N , Zhang Y, Su S, Feng Y, Wang B, Li Z. Exposure characteristics of legacy and novel per- and polyfluoroalkyl substances in blood and association with hypertension among low-exposure population. J Hazard Mater 2023;459:132185. 37531760 10.1016/j.jhazmat.2023.132185

[hoaf044-B25] Luo K , Liu X, Nian M, Wang Y, Qiu J, Yu H, Chen X, Zhang J; Shanghai Birth Cohort. Environmental exposure to per- and polyfluoroalkyl substances mixture and male reproductive hormones. Environ Int 2021;152:106496. 33744484 10.1016/j.envint.2021.106496

[hoaf044-B26] Mao D , Ding G, Wang Z, Zhao J, Li H, Lei X, Zheng J, Zhang Y, Shi R, Yuan T et al Associations of legacy perfluoroalkyl and polyfluoroalkyl substances, alternatives, and isomers with gestational diabetes mellitus and glucose homeostasis among women conceiving through assisted reproduction in Shanghai, China. Environ Sci Pollut Res Int 2024;31:14088–14102. 38273080 10.1007/s11356-023-31605-2

[hoaf044-B27] Neff AM , Laws MJ, Warner GR, Flaws JA. The effects of environmental contaminant exposure on reproductive aging and the menopause transition. Curr Environ Health Rep 2022;9:53–79. 35103957 10.1007/s40572-022-00334-y PMC8988816

[hoaf044-B28] Nian M , Luo K, Luo F, Aimuzi R, Huo X, Chen Q, Tian Y, Zhang J. Association between prenatal exposure to PFAS and fetal sex hormones: are the short-chain PFAS safer? Environ Sci Technol 2020;54:8291–8299. 32525661 10.1021/acs.est.0c02444

[hoaf044-B29] Rodríguez-Carrillo A , Remy S, Koppen G, Wauters N, Freire C, Olivas-Martínez A, Schillemans T, Åkesson A, Desalegn A, Iszatt N et al PFAS association with kisspeptin and sex hormones in teenagers of the HBM4EU aligned studies. Environ Pollut 2023;335:122214. 37482334 10.1016/j.envpol.2023.122214

[hoaf044-B30] Sang Q , Ray PF, Wang L. Understanding the genetics of human infertility. Science 2023;380:158–163. 37053320 10.1126/science.adf7760

[hoaf044-B31] Shi Z , Zhang H, Ding L, Feng Y, Xu M, Dai J. The effect of perfluorododecanonic acid on endocrine status, sex hormones and expression of steroidogenic genes in pubertal female rats. Reprod Toxicol 2009;27:352–359. 19429406 10.1016/j.reprotox.2009.02.008

[hoaf044-B32] Spyrakis F , Dragani TA. The EU’s per- and polyfluoroalkyl substances (PFAS) ban: a case of policy over science. Toxics 2023;11:721. 37755732 10.3390/toxics11090721 PMC10536631

[hoaf044-B33] Szeliga A , Calik-Ksepka A, Maciejewska-Jeske M, Grymowicz M, Smolarczyk K, Kostrzak A, Smolarczyk R, Rudnicka E, Meczekalski B. Autoimmune diseases in patients with premature ovarian insufficiency-our current state of knowledge. Int J Mol Sci 2021;22:2594. 33807517 10.3390/ijms22052594 PMC7961833

[hoaf044-B34] Wasel O , Thompson KM, Freeman JL. Assessment of unique behavioral, morphological, and molecular alterations in the comparative developmental toxicity profiles of PFOA, PFHxA, and PFBA using the zebrafish model system. Environ Int 2022;170:107642. 36410238 10.1016/j.envint.2022.107642 PMC9744091

[hoaf044-B35] Zegers-Hochschild F , Adamson GD, Dyer S, Racowsky C, de Mouzon J, Sokol R, Rienzi L, Sunde A, Schmidt L, Cooke ID et al The International Glossary on Infertility and Fertility Care, 2017. Hum Reprod 2017;32:1786–1801. 29117321 10.1093/humrep/dex234 PMC5850297

[hoaf044-B36] Zhang C , Yu D, Mei Y, Liu S, Shao H, Sun Q, Lu Q, Hu J, Gu H. Single-cell RNA sequencing of peripheral blood reveals immune cell dysfunction in premature ovarian insufficiency. Front Endocrinol (Lausanne) 2023a;14:1129657. 37223018 10.3389/fendo.2023.1129657 PMC10200870

[hoaf044-B37] Zhang S , Tan R, Pan R, Xiong J, Tian Y, Wu J, Chen L. Association of perfluoroalkyl and polyfluoroalkyl substances with premature ovarian insufficiency in Chinese women. J Clin Endocrinol Metab 2018;103:2543–2551. 29986037 10.1210/jc.2017-02783

[hoaf044-B38] Zhang Z , Tian J, Liu W, Zhou J, Zhang Y, Ding L, Sun H, Yan G, Sheng X. Perfluorooctanoic acid exposure leads to defect in follicular development through disrupting the mitochondrial electron transport chain in granulosa cells. Sci Total Environ 2023b;905:166954. 37722425 10.1016/j.scitotenv.2023.166954

[hoaf044-B39] Zhao M , Yao Y, Dong X, Baqar M, Fang B, Chen H, Sun H. Nontarget identification of novel per- and polyfluoroalkyl substances (PFAS) n soils from an oil refinery in southwestern China: a combined approach with TOP assay. Environ Sci Technol 2023;57:20194–20205. 37991390 10.1021/acs.est.3c05859

[hoaf044-B40] Zhou W , Zhang L, Tong C, Fang F, Zhao S, Tian Y, Tao Y, Zhang J; Shanghai Birth Cohort Study. Plasma perfluoroalkyl and polyfluoroalkyl substances concentration and menstrual cycle characteristics in preconception women. Environ Health Perspect 2017;125:067012. 28657892 10.1289/EHP1203 PMC5743639

