# Supplementary material for: Association between novel per- and poly-fluoroalkyl substances and premature ovarian insufficiency: a case–control study
Source: Hum Reprod Open. 2025 Jul 12;2025(3):hoaf044. doi: 10.1093/hropen/hoaf044 (PMC12308182; doi:10.1093/hropen/hoaf044)
Supplement: hoaf044_Supplementary_Data [file hoaf044_supplementary_data.docx]

**Supplementary Material**

**Association between Novel Per-and Polyfluoroalkyl Substances and Premature Ovarian Insufficiency: A Case-Control Study**

Running title: Novel PFAS and Risk of Premature Ovarian Insufficiency

Rui Qiao^1,2†^, Fanghao Guo^1,2†^, Haixia Ding^1,2†^, Di Sun^1,2^, Qianhui Hu^1,2^, Yanquan Li ^1,2^, Meiling Zhang^1,2*^, Qing Zhang^1,2*^, Wen Li^1,2*^

^1^ The International Peace Maternity and Child Health Hospital, School of Medicine, Shanghai Jiao Tong University, Shanghai 200030, China

^2^ Shanghai Key Laboratory of Embryo Original Diseases, Shanghai 200030, China

**Correspondence address:**

Meiling Zhang: Center for Reproductive Medicine & Fertility Preservation Program, International Peace Maternity and Child Health Hospital, School of Medicine, Shanghai Jiao Tong University, No. 910 Hengshan Road, Xuhui District, Shanghai, 200030, China. Email: zhml1119@sina.com, ORCID: <https://orcid.org/0000-0003-1256-5511>

Qing Zhang: Center for Reproductive Medicine & Fertility Preservation Program, International Peace Maternity and Child Health Hospital, School of Medicine, Shanghai Jiao Tong University, No. 910 Hengshan Road, Xuhui District, Shanghai, 200030, China. Email: zhangqing081@sina.com, ORCID: https://orcid.org/0000-0001-5190-7751

Wen Li: Center for Reproductive Medicine & Fertility Preservation Program, International Peace Maternity and Child Health Hospital, School of Medicine, Shanghai Jiao Tong University, No. 910 Hengshan Road, Xuhui District, Shanghai, 200030, China. Email: [liwen@shsmu.edu.cn](mailto:liwen@shsmu.edu.cn), ORCID: https://orcid.org/0000-0001-9468-5570

† These authors contributed equally to this work.

Supplementary Table S1. The concentration ranges of exposure levels for each chemical in all participants.

Supplementary Table S2. Association between novel PFAS concentrations and POI (age < 35y).

Supplementary Table S3. Association between novel PFAS concentrations and POI (age > 35y).

Supplementary Table S4. Unconditional logistic regression models for the associations of novel PFAS with POI: a case-control study conduct in Shanghai during 2023–2024 (n = 371).

Supplementary Figure S1. Directed Acyclic Graph for covariate selection.

Supplementary Figure S2. Heatmap of the Spearman correlation matrix of novel PFAS.

Supplementary Figure S3. Univariate exposure–response relationship between novel PFAS and POI from BKMR model (n = 371)

Supplementary Figure S4. Bivariate exposure-response associations between novel PFAS and POI from BKMR model (n = 371)

**Supplementary Table S1. The concentration ranges of exposure levels for each chemical in all participants.**

| **Compound** | **Q1** | **Q2** | **Q3** | **Q4** |
| --- | --- | --- | --- | --- |
| **PFBA** | < 0.014 | 0.014 – 0.028 | 0.028 – 0.054 | > 0.054 |
| **PFPeA** | < 0.003 | 0.003 – 0.005 | 0.005 – 0.009 | > 0.009 |
| **PFBS** | < 0.072 | 0.072 – 0.111 | 0.111 – 0.184 | > 0.184 |
| **PFPeS** | < 0.007 | 0.007 – 0.011 | 0.011 – 0.017 | > 0.017 |
| **HFPO-DA** | < 0.006 | 0.006 – 0.009 | 0.009 – 0.014 | > 0.014 |
| **6:2 ClPFESA** | < 0.852 | 0.852 – 1.632 | 1.632 – 3.202 | > 3.202 |

Note: The unit for each chemical is ng/ml.

Concentration ranges were measured according to the quartile of each chemical in all participants.

**Supplementary Table S2. Association between Novel PFAS concentrations and POI (age < 35y).**

| **Compound^#^** | **Crude OR** | **Crude OR *P*- Value** | **Adjusted OR^§^** | **Adjusted OR *P*- Value^§^** |
| --- | --- | --- | --- | --- |
| **PFBA** | 1.86 (1.32 – 2.63) | < 0.001 | 2.14 (1.46 – 3.14) | 0.002 |
| **PFPeA** | 3.81 (2.50 – 5.94) | < 0.001 | 4.13 (2.51 – 6.79) | < 0.001 |
| **PFBS** | 0.59 (0.37 – 0.97) | 0.037 | 0.57 (0.34 – 0.95) | 0.032 |
| **PFPeS** | 2.25 (1.24 – 4.10) | 0.007 | 2.05 (1.06 – 3.94) | 0.012 |
| **HFPO-DA** | 1.80 (1.10 – 2.94) | < 0.018 | 1.89 (1.11 – 3.21) | 0.019 |
| **6:2 ClPFESA** | 0.89 (0.63 – 1.21) | 0.887 | 0.99 (0.66 – 1.31) | 0.060 |

Note: ^#^The concentrations of Novel PFAS were natural logarithmic transformed. ^§^ Multivariate logistic regression model with adjustments of age, BMI, education, infertility type and living address.

**Supplementary Table S3. Association between Novel PFAS concentrations and POI (age > 35y).**

| **Compound^#^** | **Crude OR** | **Crude OR *P*- Value** | **Adjusted OR^§^** | **Adjusted OR *P*- Value^§^** |
| --- | --- | --- | --- | --- |
| **PFBA** | 1.38 (0.79 – 2.42) | 0.252 | 1.62 (0.86 – 3.05) | 0.137 |
| **PFPeA** | 5.36 (2.41 – 11.92) | < 0.001 | 5.59 (2.20 – 14.17) | < 0.001 |
| **PFBS** | 0.33 (0.13 – 0.85) | 0.014 | 0.29 (0.10 – 0.85) | 0.024 |
| **PFPeS** | 2.00 (0.94 – 4.27) | 0.072 | 2.52 (0.98 – 6.42) | 0.053 |
| **HFPO-DA** | 5.50 (2.26 – 13.3) | < 0.001 | 5.78 (2.03 – 16.42) | < 0.001 |
| **6:2 ClPFESA** | 1.07 (0.60 – 1.93) | 0.808 | 1.18 (0.62 – 2.24) | 0.611 |

Note: ^#^The concentrations of Novel PFAS were natural logarithmic transformed. ^§^ Multivariate logistic regression model with adjustments of age, BMI, education, infertility type and living address.

**Supplementary Table S4. Unconditional logistic regression models for the associations of Novel PFAS with POI: a-case control study conduct in Shanghai during 2023–2024 (n = 371).**

| **Compound** | **Overall**  **（*N* = 371）**  **[*n* (%)]** | | **POI cases**  **（*N* = 151）**  **[*n* (%)]** | **Controls**  **（*N* = 220）**  **[*n* (%)]** | **Crude OR** | **Crude OR**  ***P*- Value** | **Adjusted OR^§^** | **Adjusted OR**  ***P*- Value^§^** |
| --- | --- | --- | --- | --- | --- | --- | --- | --- |
| **PFBA** | | | | | | | | |
| **Q1** | | 93 (25.1) | 23 (15.2) | 70 (31.8) | 1 (ref) |  | 1 (ref) |  |
| **Q2** | | 93 (25.1) | 28 (18.5) | 65 (29.5) | 1.26 (0.58 – 2.74) | 0.559 | 1.34 (0.59 – 3.07) | 0.488 |
| **Q3** | | 93 (25.1) | 45 (29.8) | 48 (21.8) | 3.27 (1.46 – 7.35) | 0.004 | 3.17 (1.36 – 7.37) | 0.008 |
| **Q4** | | 92 (24.8) | 55 (36.4) | 37 (16.8) | 3.39 (1.43 – 8.02) | 0.006 | 3.68 (1.49 – 9.08) | 0.005 |
| **PFPeA** | | | | | | | | |
| **Q1** | | 93 (25.1) | 9 (6.0) | 84 (38.2) | 1 (ref) |  | 1 (ref) |  |
| **Q2** | | 93 (25.1) | 31 (20.5) | 62 (28.2) | 6.78 (2.75 – 16.68) | < 0.001 | 5.91 (2.33 – 15.03) | < 0.001 |
| **Q3** | | 93 (25.1) | 43 (28.5) | 50 (22.7) | 11.82 (4.78 – 29.24) | < 0.001 | 13.40 (5.25 – 34.18) | < 0.001 |
| **Q4** | | 92 (24.8) | 68 (45.0) | 24 (10.9) | 29.67 (11.81 – 74.54) | < 0.001 | 28.23 (10.75 – 74.10) | < 0.001 |
| **PFBS** | |  |  |  |  |  |  |  |
| **Q1** | | 93 (25.1) | 42 (27.8) | 51 (23.2) | 1 (ref) |  | 1 (ref) |  |
| **Q2** | | 93 (25.1) | 42 (27.8) | 51 (23.2) | 0.86 (0.40 – 1.89) | 0.711 | 0.80 (0.35 – 1.79) | 0.586 |
| **Q3** | | 93 (25.1) | 35 (23.2) | 58 (26.4) | 0.39 (0.18 – 0.88) | 0.023 | 0.36 (0.16 – 0.85) | 0.019 |
| **Q4** | | 92 (24.8) | 32 (21.2) | 60 (27.3) | 0.28 (0.12 – 0.63) | 0.002 | 0.27 (0.11 – 0.64) | 0.003 |
| **PFPeS** | |  |  |  |  |  |  |  |
| **Q1** | | 93 (25.1) | 21 (13.9) | 72 (32.7) | 1 (ref) |  | 1 (ref) |  |
| **Q2** | | 93 (25.1) | 35 (23.2) | 58 (26.4) | 1.75 (0.80 – 3.83) | 0.164 | 1.70 (0.74 – 3.92) | 0.213 |
| **Q3** | | 93 (25.1) | 48 (31.8) | 45 (20.5) | 3.61 (1.61 – 8.07) | 0.002 | 4.11 (1.76 – 9.62) | 0.001 |
| **Q4** | | 92 (24.8) | 47 (31.1) | 45 (20.5) | 3.11 (1.35 – 7.19) | 0.008 | 3.81 (1.57 – 9.28) | 0.003 |
| **HFPO-DA** | |  |  |  |  |  |  |  |
| **Q1** | | 93 (25.1) | 23 (15.2) | 70 (31.8) | 1 (ref) |  | 1 (ref) |  |
| **Q2** | | 93 (25.1) | 29 (19.2) | 64 (29.1) | 1.24 (0. 58 – 2.63) | 0.061 | 1.11 (0.50 – 2.46) | 0.799 |
| **Q3** | | 93 (25.1) | 35 (23.2) | 58 (26.4) | 1.31 (0.60 – 2.88) | 0.009 | 1.20 (0.54 – 2.71) | 0.654 |
| **Q4** | | 92 (24.8) | 64 (42.4) | 28 (12.7) | 5.34 (2.40 – 11.92) | < 0.001 | 5.10 (2.21 – 11.78) | < 0.001 |
| **6:2 ClPFESA** | |  |  |  |  |  |  |  |
| **Q1** | | 93 (25.1) | 38 (25.2) | 55 (25.0) | 1 (ref) |  | 1 (ref) |  |
| **Q2** | | 93 (25.1) | 42 (27.8) | 51 (23.2) | 1.00 (0.47 – 2.15) | 0.992 | 0.93 (0.42 – 2.08) | 0.858 |
| **Q3** | | 93 (25.1) | 35 (23.2) | 58 (26.4) | 0.81 (0.38 – 1.72) | 0.577 | 0.76 (0.34 – 1.70) | 0.511 |
| **Q4** | | 92 (24.8) | 36 (23.8) | 56 (25.5) | 0.74 (0.34 – 1.61) | 0.449 | 0.71 (0.31 – 1.63) | 0.424 |

The concentrations of Novel PFAS were categorized into four levels (Q1, Q2, Q3, and Q4) according to their tertiles calculated based on all participants. Detailed information about the tertile definitions is presented in Supplementary Table S1. ^§^ Multivariate logistic regression model with adjustments of age, BMI, education, infertility type and living address.

**
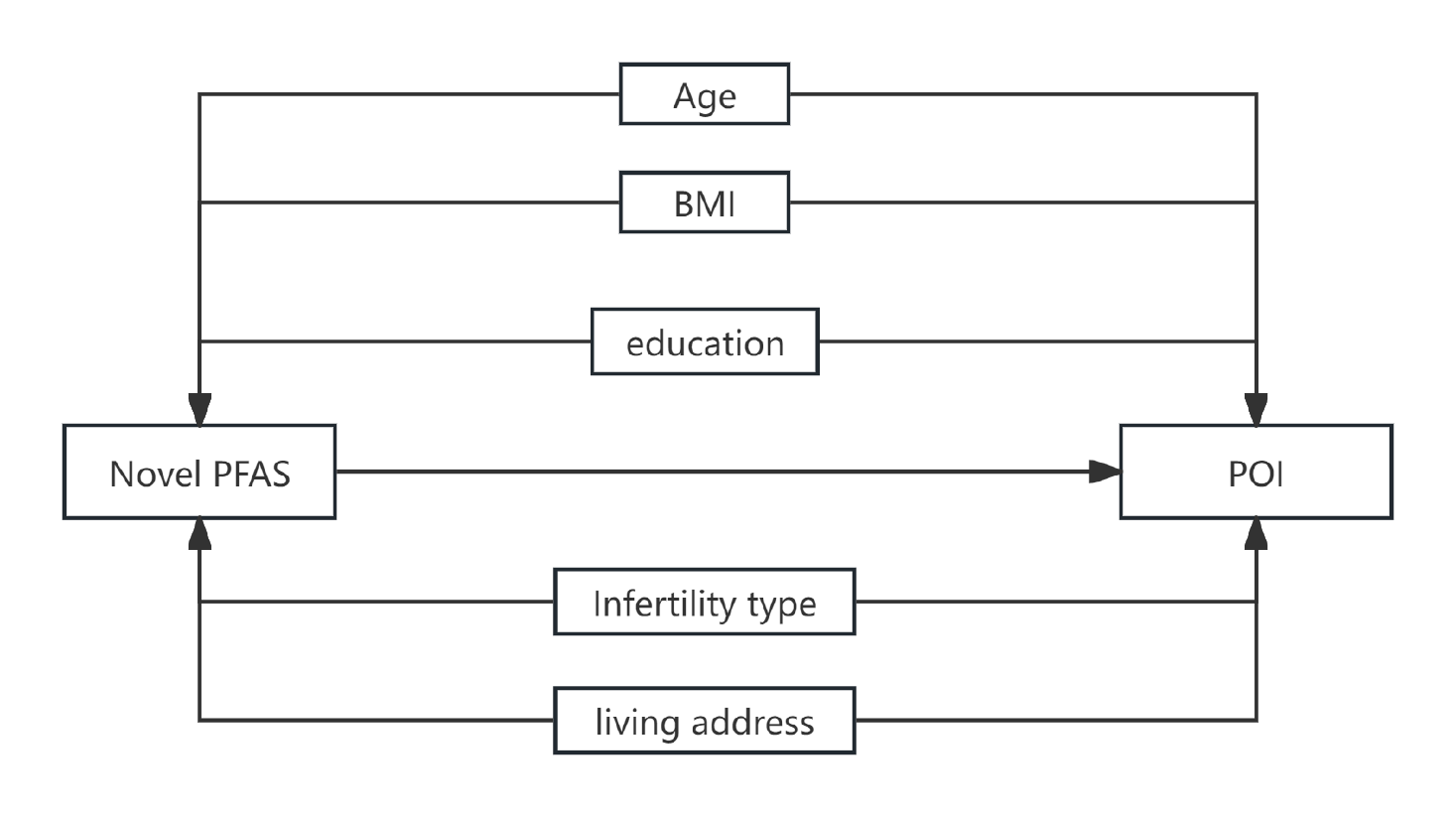
**

**Supplementary Figure. S1** Directed Acyclic Graph for covariate selection. POI, Premature Ovarian Insufficiency.

**
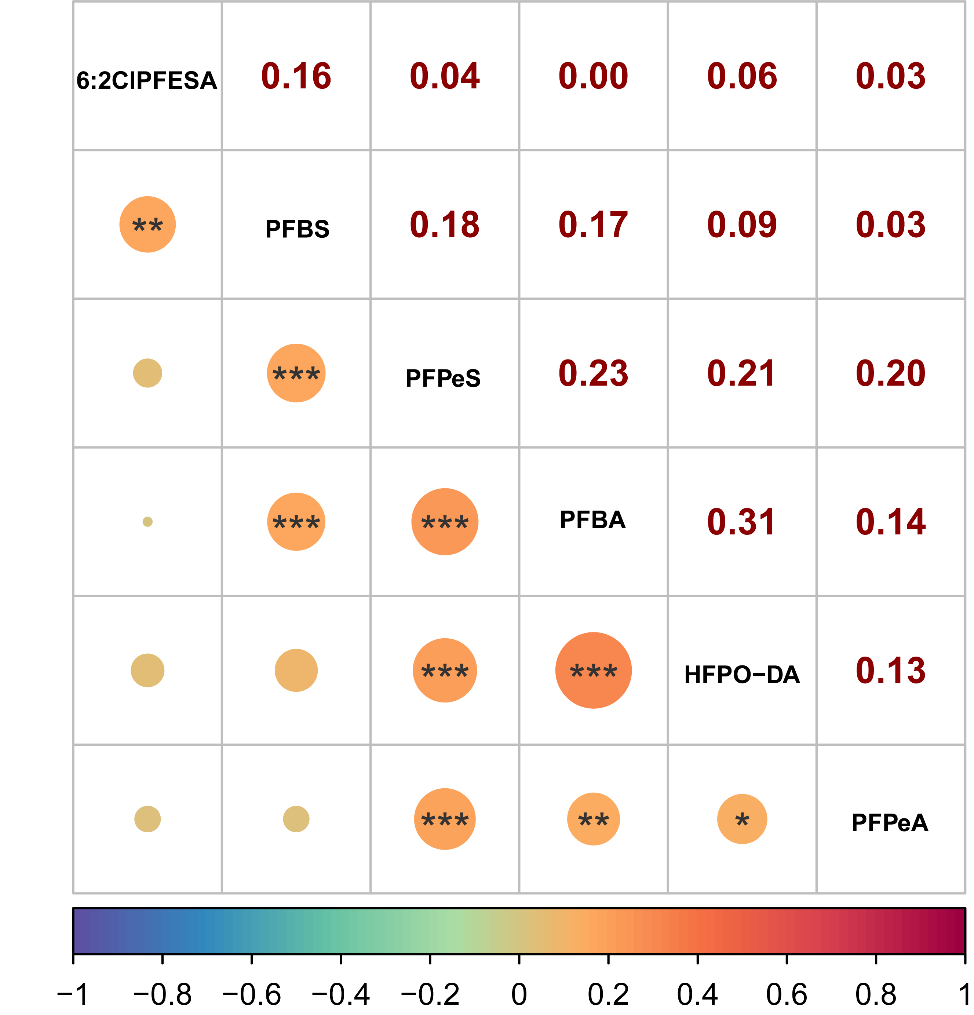
**

**Supplementary Figure S2.** Clustered heatmap of the Pearson correlation matrix of Novel PFAS (ln-transformed) (n = 371). The circles and numbers in the box are pairwise correlation coefficients. For full chemical names see Table 1. Note: **P* < 0.05, ***P* < 0.01, ****P* < 0.001.

**
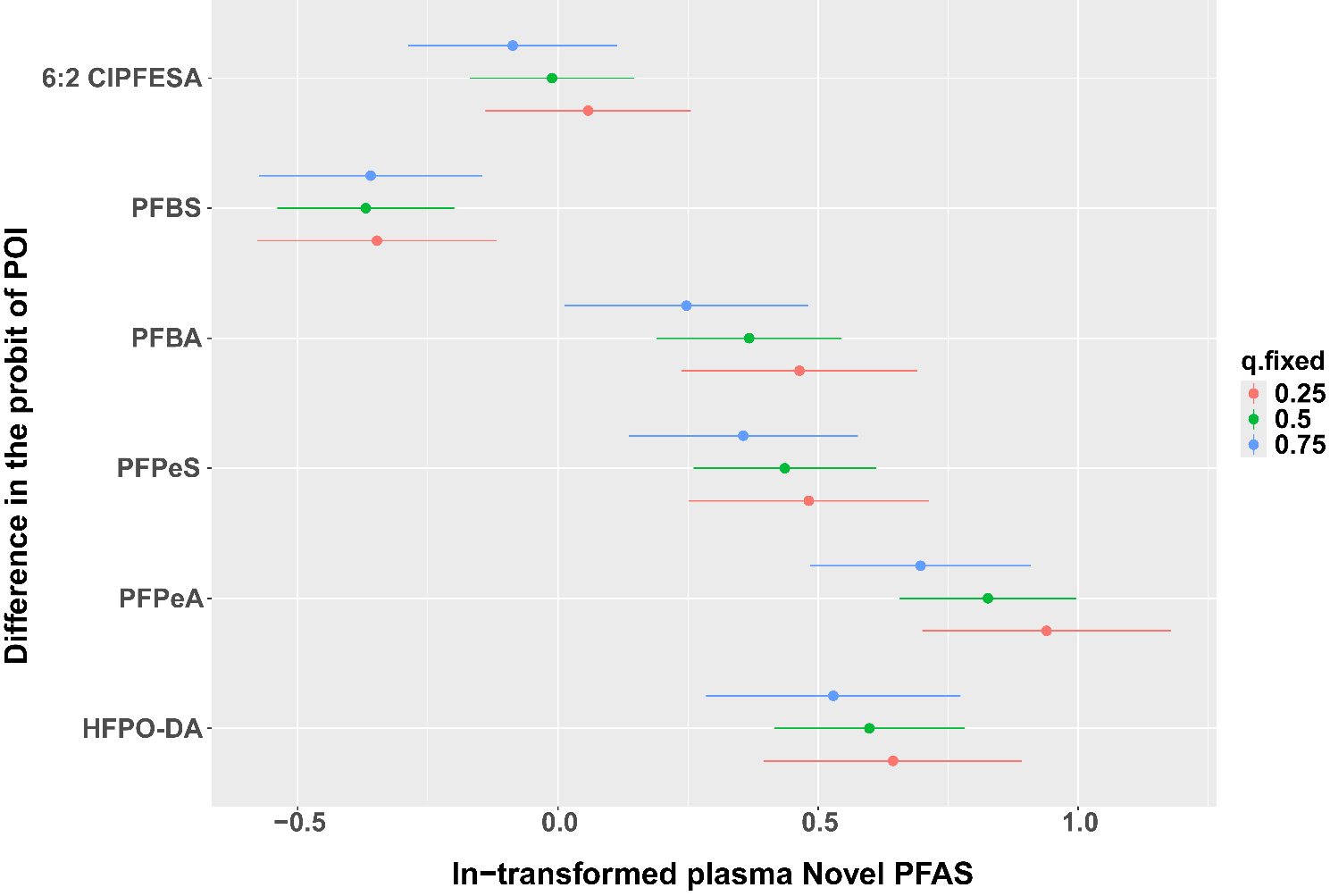
**

**Supplementary Figure S3.** Univariate exposure–response relationship of individual plasma Novel PFAS concentrations (estimates and 95% CIs) in Chinese women diagnosed with POI estimated by Bayesian kernel machine regression (BKMR) for each Novel PFAS, with the other pollutants fixed at the median (n = 371). All estimates were adjusted for age, BMI, education, infertility type and living address. The boundaries of the gray areas represent the 95% CIs of the exposure–response relationship. For full chemical names see Table 1. Note: BMI, body mass index; POI, Premature Ovarian Insufficiency; PFAS, per- and polyfluoroalkyl substances.

**
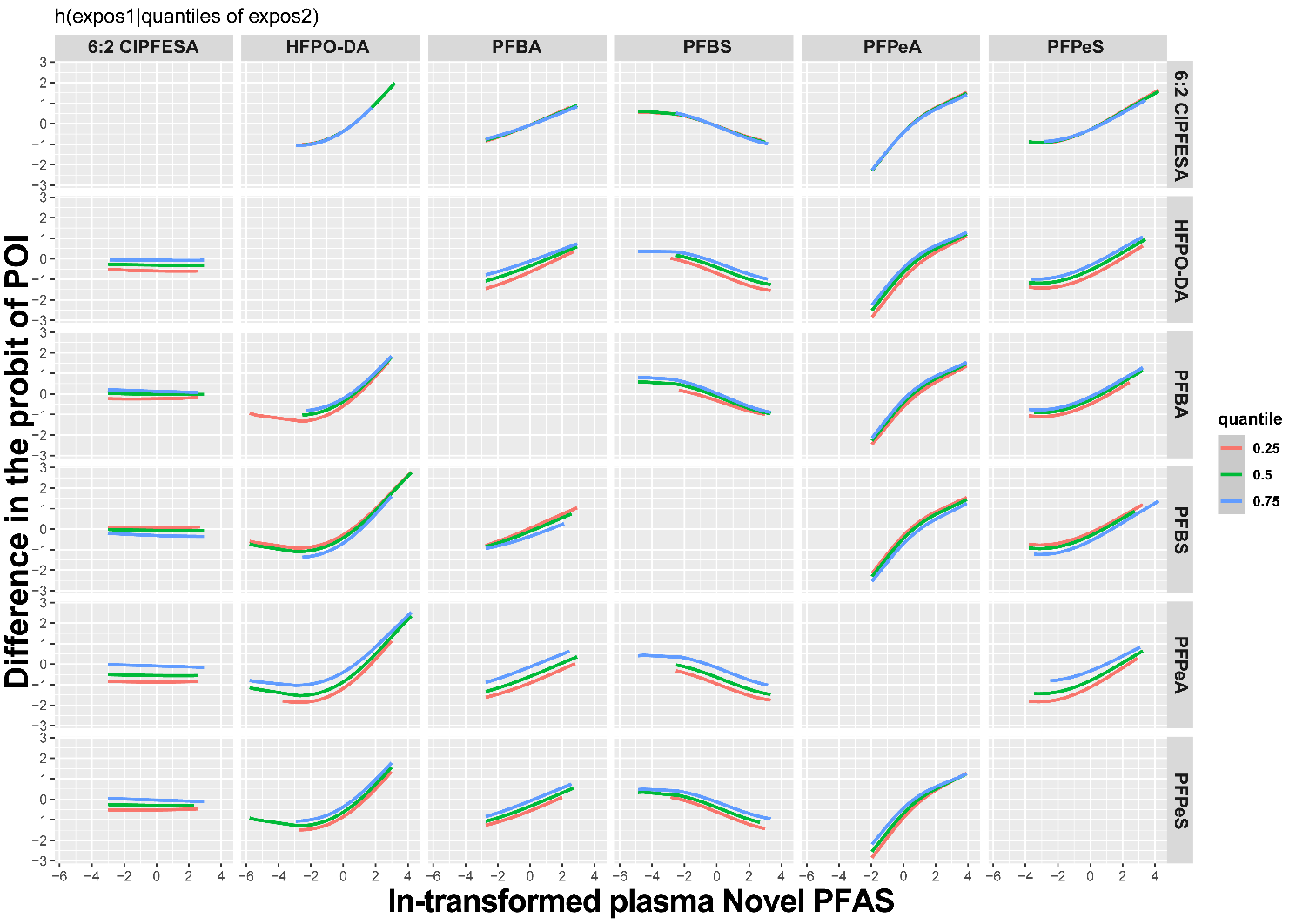
**

**Supplementary Figure S4.** Bivariate exposure-response associations between Novel PFAS and POI from BKMR model (n = 371). All estimates were adjusted for age, BMI, education level, infertility type and living address. Bivariate exposure-response functions or one PFAS when another PFAS fixed at either the 25th, 50th, or 75th percentile and the remaining Novel PFAS are fixed at the median. For full chemical names see Table 1.
